# Breastfeeding duration and brain-body development in 9–10-year-olds: modulating effect of socioeconomic levels

**DOI:** 10.1038/s41390-024-03330-0

**Published:** 2024-06-15

**Authors:** Vidya Rajagopalan, Eustace Hsu, Shan Luo

**Affiliations:** 1 Division of Cardiology, Department of Pediatrics, Children’s Hospital Los Angeles, Keck School of Medicine, University of Southern California, Los Angeles, CA USA; 2 Division of Endocrinology and Diabetes, Department of Medicine, Keck School of Medicine, University of Southern California, Los Angeles, CA USA; 3 Diabetes and Obesity Research Institute, Keck School of Medicine, University of Southern California, Los Angeles, CA USA; 4 Department of Psychology, University of Southern California, Los Angeles, CA USA; 5 Center for Endocrinology, Diabetes and Metabolism, Children’s Hospital Los Angeles, Los Angeles, CA USA; 6 Neuroscience Graduate Program, University of Southern California, Los Angeles, CA USA

## Abstract

**Objective:**

To investigate relationships of breastfeeding duration with brain structure and adiposity markers in youth and how these relationships are modified by neighborhood socioeconomic environments (SEEs).

**Methods:**

This was a cross-sectional study of youth enrolled in the Adolescent Brain and Cognitive Development (ABCD) Study® (*n* = 7511). Mixed effects models examined associations of breastfeeding duration with global brain measures and adiposity markers, adjusting for sociodemographic, pre- and post-natal covariates. Stratified analysis was performed by area deprivation index (ADI) tertiles.

**Results:**

Total cortical surface area (SA) (False Discovery Rate - FDR corrected *P* < 0.001), cortical (FDR corrected *P* < 0.001) and subcortical gray matter (GM) volume (FDR corrected *P* < 0.001) increased with increased breastfeeding duration. Body mass index (BMI) *z*-scores (FDR corrected *P* = 0.001), waist circumference (FDR corrected *P* = 0.002) and waist-to-height ratio (WHtR) (FDR corrected *P* = 0.001) decreased with increased breastfeeding duration. Breastfeeding duration was inversely associated with adiposity in youth from high- and medium- ADI neighborhoods, but positively associated with SA across ADI tertiles.

**Conclusions:**

In this cross-sectional study, longer breastfeeding duration was associated with lower adiposity indices, particularly in youth from lower SEEs and greater SA across SEE levels. Longer breastfeeding duration showed long-term associations with brain and body development for offspring.

**Impact:**

- Building on previous findings that longer breastfeeding duration is associated with healthier weight gain, lower obesity risk, and brain white matter development in infancy, our results find longer breastfeeding duration to be associated with lower adiposity indices and greater cortical and subcortical gray matter volume, and cortical surface area during peri-adolescence.
- Children from lower socioeconomic environments (SEEs) demonstrated stronger negative associations of breastfeeding duration and adiposity indices, and children across SEEs showed positive relationships between breastfeeding duration and cortical surface area.
- Promoting breastfeeding, particularly among women from lower SEEs would confer long-term benefits to offspring.

## Introduction

The short-term, multi-faceted benefits of breastfeeding for offspring brain and body development have been established. It is known that longer breastfeeding duration correlates with healthy weight gain and lower rates of infant obesity.^1^ Additionally, multiple studies in children at various ages have shown that those who were breastfed longer exhibit white (WM) and gray matter (GM) differences in the brain^2–7^ that are considered desirable because of its strong association with better neurodevelopmental outcomes.^8–10^ But there is limited knowledge on if these associations persist into late childhood.^7^

Although there is emerging evidence showing long-term associations of breastfeeding with brain and body development in youth, they were limited by (a) the use of proxies such as obesity incidence and school performance and (b) limited sample size of a largely homogenous population. In addition to not being informative about underlying biological processes, these proxies are also strongly influenced by socioeconomic factors. Socioeconomic environment (SEE) is an important influencer of child development with higher SEE levels correlated with lower adiposity indices and greater brain structural measures.^11–13^ Furthermore, studies tend to overstate long-term effects of breastfeeding because of selection bias towards high SEE families who are more likely to breastfeed longer.^14–16^ It remains unclear if there exist long-term associations of breastfeeding with brain and body development and if these associations vary by SEEs.

In this study, we leveraged demographic, gestational and infant health history, neighborhood SEE, anthropometric and brain data from the Adolescent Brain Cognitive Development (ABCD) study, the largest long-term study of pediatric brain development in the U.S., to determine relationships of breastfeeding duration with global brain structure and adiposity markers in a cohort of 7511 9–10-year-olds. Using these measures, as opposed to obesity incidence or test scores, we can better investigate the underlying biological changes associated with breastfeeding. We focused on global vs. regional brain measures in this study given prior reports indicating a consistent pattern of longer breastfeeding duration and larger global brain measures years later^2,3,8^ but inconsistent results for regional brain measures.^4–6,17,18^ The current study aims to investigate long-term associations between breastfeeding duration in infancy and brain structure and adiposity indices in youth across SEE groups. We hypothesize that duration of breastfeeding will have an inverse relationship with adiposity measures and positive relationship with global brain measures (e.g., volumes, cortical thickness, surface area). Based on prior work, we anticipate that these associations will be more pronounced in the higher SEE groups.^19,20^

## Methods

### Participants

Data was obtained from the baseline assessments of ABCD 3.0 data release (*N* = 11,875). Details of ABCD study design, recruitment and inclusion/exclusion were mentioned elsewhere.^21^ In short, ABCD study recruitment was completed between September 2016 and August 2018 and baseline data were collected between September 2016 and October 2018. Participants with missing data for any study-specific variables were excluded. After applying study-specific exclusion criteria on anthropometric measurements, neuroimaging, and other covariates as described in the next sections, our final sample size is 7511. Parents gave written informed consent and youth, including siblings, provided assent to take part in the ABCD Study. The Institutional Review Board at the University of Southern California approved the current study. This study followed the Strengthening the Reporting of Observational Studies in Epidemiology (STROBE) reporting guideline for cohort studies.^22^

### Breastfeeding duration

Breastfeeding duration was self-reported by the caregiver as an integer number of months. 88.8% of breastfeeding duration data were provided by the participant’s biological mother, 9.6% by the participant’s biological father, 0.3% by an adoptive parent, 0.4% by a custodial parent, and 0.9% by another caregiver. Reported breastfeeding duration was coded into 4 subcategories: 0 months, 1–6 months, 7–12 months, >12 months. Categorizations were made with consideration to guidelines from the American Academy of Pediatrics, for synchronization with cut points used elsewhere in the literature, and to ensure adequate sample sizes within subgroupings.^23–26^ The category of 0 months also includes those who may have initiated breastfeeding but weaned before 1 month because non-integer answers were not possible. The questionnaires measured any breastfeeding not just exclusive breastfeeding (in the first 6 months) and did not distinguish between direct breastfeeding and breastmilk feeding.

### Neighborhood socioeconomic environment

Area Deprivation Index (ADI) was used to measure neighborhood SEE. ADI is a combination of characteristics of a neighborhood reported from the American Community Survey (2011-2015),^27,28^ with higher ADI score representing lower SEE. For this study, ADI was computed using caregiver-provided current addresses, which were matched with 2010 US census tracts to identify neighborhoods of residence.^29^ ADI percentile score was computed for each participant corresponding to their primary residence, then was grouped into tertiles for ease of interpretation.^29^

### Adiposity markers

Weight, height, and waist circumference were recorded by a trained researcher at baseline visit. Waist circumference measurements were taken with a tape measure along the highest point of the pelvic bone.^30^ Height and weight were recorded as the mean of up to three measurements.^31^ All measurements were converted to metric scale and used to calculate waist-to-height ratio (WHtR) and body mass index (BMI, kg/m^2^).

Measurements were further converted into age- and sex- specific BMI percentiles, BMI *z*-scores, weight *z*-scores, height *z*-scores and waist *z*-scores.^32–34^ The following criteria were used to exclude implausible anthropometrics data following procedures used in previous studies: (1) BMI *z*-scores ≤ –4 SDs or ≥8 SDs,^31,35^ (2) BMI < 10^36^, (3) weight *z*-scores ≤–5 SDs or ≥8 SDs,^35^ (4) height *z*-scores <–5 SDs or >4 SDs,^35^ (5) waist *z*-scores <–4 SDs or >4 SDs,^30^ (6) WHtR ≤0.3 or ≥1.^37^

### Neuroimaging

T1-weighted anatomical scans were collected using methods optimized for 3-T scanners across multiple platforms at 21 ABCD Study sites.^38,39^ Neuroimaging quality control procedures were implemented at the ABCD data analytics and informatics center, consisting of evaluation of images on five categories of inaccuracy: severity of motion, intensity inhomogeneity, white matter underestimation, pial overestimation, and magnetic susceptibility artifact. Participants were excluded for analysis for failure to pass quality control on any category, as well for abnormal radiological findings. T1 Images that passed ABCD quality control were segmented using FreeSurfer (version 5.3.0), yielding the following measures according to the Desikan-Killiany Atlases^40^: total volumes of cortical gray, subcortical gray (GM) and white matter (WM) (mm^3^), cortical thickness (mm), and cortical surface area (SA) (mm^2^).^41^

### Covariates

The following variables were extracted from the ABCD database: gestational age, child health problems at birth, current age, sex assigned at birth, pubertal stage, race/ethnicity, parental education, family income, maternal age, maternal health problems during pregnancy, and maternal alcohol or tobacco use during pregnancy. Race/ethnicity was self-reported by caregivers and grouped into five categories: Asian, Black non-Hispanic, Hispanic or Latino, White non-Hispanic, and Other, where “Other” included youth identified as Native American or American Indian, Native Hawaiian, Pacific Islander (Guamanian, Samoan, or other Pacific Islander), and multiple races (Asian, non-Hispanic Black, non-Hispanic White). Parental education was coded as a binary variable indicating whether at least one parent has obtained a bachelor’s degree. Yearly household income places combined income into three categories: less than $50,000, between $50,000 and $99,999, and at least $100,000.^42,43^ Further details can be found in Supporting Methods.

### Data analysis

All analyses were conducted in R (R version 4.0.3).^44^ Linear mixed effects models were used to examine correlations between breastfeeding duration (independent variable) and each global brain measurement and adiposity marker (dependent variable) for the whole cohort, while controlling for covariates known to influence brain structure and adiposity markers.^45–56^ Age and sex were not included as covariates for models with BMI *z*-scores as a dependent variable. Models of brain measurements included handedness, and intracranial volume for volumetric measures. Family ID, nested within site, were modeled as random intercepts in models of adiposity markers; Family ID, nested within scanner ID, were modeled as random intercepts for models of brain measurements, to account for shared variation related to shared family membership, study visit location, and/or scanner.

We investigated the interaction of ADI and breastfeeding duration only on those brain and adiposity measures identified as significant in the whole cohort model, followed by stratified analysis by each ADI tertile.

Linear mixed effects models were performed using the *lme4* package.^57^ Standardized betas and 95% confidence intervals were reported. Standardized betas represent standard deviations increase in dependent variable corresponding to each unit increase in breastfeeding duration category. *P*-values were calculated using Satterthwaite’s method in the *lmerTest* package,^58^ and tests of significance (2-tailed) were corrected for multiple comparisons using the Benjamini-Hochberg false discovery rate (FDR), with *P* < 0.05 as the corrected threshold for significance.

## Results

### Participant Demographics

The final sample included 7511 youth (mean [SD] age, 9.92 [0.6] years; 3881 males [52%]; 1437 Hispanic individuals [19%], 4308 White individuals [57%], 877 Black individuals [12%], 129 Asian individual [2%] and 760 Other individuals [10%]). Table 1 describes characteristics of the sample by breastfeeding duration category. 18.8% of child participants were breastfed for 0 months, 35.3% for 1–6 months, 24.9% for 7–12 months, and 21.0% for greater than 12 months. The self-reported breastfeeding rates are comparable to CDC-reported national percentages for infants born within the same timeframe as the ABCD cohort.^59^ Participants who breastfed longer were older in gestational age (*F*_3,7507_ = 67.00, *P* < 0.001), healthier at birth (Χ^2^_3_ = 20.85, *P* < 0.001), younger at the time of study visit (*F*_3,7507_ = 6.77, *P* < 0.001), less advanced in current pubertal status (Χ^2^_6_ = 200.79, *P* < 0.001), more White or Asian (Χ^2^_12_ = 586.52, *P* < 0.001), higher in current family income (Χ^2^_6_ = 493.92, *P* < 0.001), higher in current parental education (Χ^2^_3_ = 767.79, *P* < 0.001), older in maternal age at birth (*F*_3,7507_ = 113.83, *P* < 0.001), less exposed to maternal health problems during pregnancy (Χ^2^_3_ = 127.84, *P* < 0.001) and maternal alcohol or tobacco use during pregnancy (Χ^2^_3_ = 28.50, *P* < 0.001*)*.

**Table 1:** Sample characteristics by breastfeeding duration category.

	No breastfeeding (N = 1412)	1–6 months (N = 2651)	7–12 months (N = 1873)	> 12 months (N = 1575)	Total (N = 7511)	*P*-value*
**Child**						
Gestational Age (months)	38.832 (2.426)	38.788 (2.466)	38.391 (1.725)	39.586 (1.423)	39.114 (2.130)	**<0.001**
Health Problems at Birth						**<0.001**
No	1046 (74.1%)	1843 (69.5%)	1375 (73.4%)	1187 (75.4%)	5451 (72.6%)	
Yes	366 (25.9%)	808 (30.5%)	498 (26.6%)	388 (24.6%)	2060 (27.4%)	
Current age (years)	9.928 (0.607)	9.952 (0.628)	9.882 (0.631)	9.878 (0.632)	9.915 (0.626)	**<0.001**
Sex						0.61
Female	667 (47.2%)	1307 (49.3%)	897 (47.9%)	759 (48.2%)	3630 (48.3%)	
Male	745 (52.8%)	1344 (50.7%)	976 (52.1%)	816 (51.8%)	3881 (51.7%)	
Puberty						**<0.001**
Pre-Puberty	609 (43.1%)	1281 (48.3%)	1112 (59.4%)	942 (59.8%)	3944 (52.5%)	
Early Puberty	312 (22.1%)	659 (24.9%)	428 (22.9%)	352 (22.3%)	1751 (23.3%)	
Mid-Post Puberty	491 (34.8%)	711 (26.8%)	333 (17.8%)	281 (17.8%)	1816 (24.2%)	
Race/Ethnicity						**<0.001**
White	606 (42.9%)	1425 (53.8%)	1212 (64.7%)	1065 (67.6%)	4308 (57.4%)	
Black	389 (27.5%)	318 (12.0%)	114 (6.1%)	56 (3.6%)	877 (11.7%)	
Hispanic	263 (18.6%)	586 (22.1%)	323 (17.2%)	265 (16.8%)	1437 (19.1%)	
Asian	8 (0.6%)	44 (1.7%)	38 (2.0%)	39 (2.5%)	129 (1.7%)	
Other**	146 (10.3%)	278 (10.5%)	186 (9.9%)	150 (9.5%)	760 (10.1%)	
**Caregiver**						
Current family income						**<0.001**
<$50,000	661 (46.8%)	751 (28.3%)	359 (19.2%)	278 (17.7%)	2049 (27.3%)	
≥$50,000 and <$100,000	419 (29.7%)	754 (28.4%)	519 (27.7%)	478 (30.3%)	2170 (28.9%)	
≥$100,000	332 (23.5%)	1146 (43.2%)	995 (53.1%)	819 (52.0%)	3292 (43.8%)	
Current Parental Education						**<0.001**
No Bachelor’s Degree	900 (63.7%)	1042 (39.3%)	445 (23.8%)	324 (20.6%)	2711 (36.1%)	
Bachelor’s Degree or Higher	512 (26.3%)	1609 (60.7%)	1428 (76.2%)	1251 (79.4%)	4800 (63.9%)	
Maternal Age at Birth(years)	27.381 (6.514)	29.547 (6.219)	30.482 (5.481)	31.140 (5.445)	29.707 (6.077)	**<0.001**
Maternal health problems during pregnancy						**<0.001**
No	760 (53.8%)	1547 (58.4%)	1277 (68.2%)	1102 (70.0%)	4686 (62.4%)	
Yes	652 (46.2%)	1104 (41.6%)	596 (31.8%)	473 (30.0%)	2825 (37.6%)	
Maternal alcohol or tobacco use during pregnancy						**<0.001**
No	898 (63.6%)	1695 (63.9%)	1322 (70.6%)	1070 (67.9%)	4985 (66.4%)	
Yes	514 (36.4%)	956 (36.1%)	551 (29.4%)	505 (32.1%)	2526 (33.6%)	

Data are mean ± SD or n (%). *Categorical variables by Χ^2^ test, continuous variables by *t*-test. Boldface indicates significance at the threshold of *P* < 0.05. **Other includes participants identified by caregiver as Native Hawaiian, Pacific Islander, Alaskan Native, American Indian or multiple races, excluding participants identified as Hispanic or Latino ethnicity.

### Breastfeeding duration and child global brain measurements

**Fig. 1:**
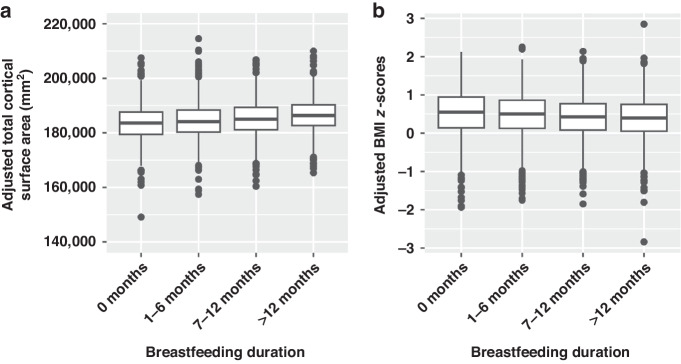
Relationships between breastfeeding duration and brain and adiposity markers. **A** Boxplots display distributions of total cortical surface area (adjusted for family ID nested within scanner ID, handedness, age, sex, pubertal status, race/ethnicity, family income, parental education, gestational age, maternal health problems during pregnancy, child health problems at birth, maternal age at birth, and maternal alcohol or tobacco use during pregnancy) separated by breastfeeding duration category. Adjusted values were computed as the sum of least-squares means and model residuals. **B** Boxplots display distributions of BMI z-scores (adjusted for family ID nested within site, pubertal status, race/ethnicity, family income, parental education, gestational age, maternal health problems during pregnancy, child health problems at birth, maternal age at birth, and maternal alcohol or tobacco use during pregnancy) separated by breastfeeding duration category.

One category increase in breastfeeding duration was associated with 947.944 mm^2^ increases in total cortical SA (β (95% CI) = 0.053 (0.033, 0.074), FDR corrected *P* < 0.001, Fig. 1A), 1180.294 mm^3^ increases in cortical GM volume (β (95% CI) = 0.021 (0.010, 0.032), FDR corrected *P* < 0.001), 80.476 mm^3^ increases in subcortical GM volume (β (95% CI) = 0.016 (0.003, 0.030), FDR corrected *P* = 0.03) and 1064.882 mm^3^ decreases in WM volume (β (95% CI) = –0.022 (–0.033, –0.011), FDR corrected *P* < 0.001) (Table 2, Fig. S1A).

**Table 2:** Associations between breastfeeding duration category and global brain measurements and adiposity markers.

	Overall (*N* = 7511)		High ADI (*N* = 2451)		Medium ADI (*N* = 2473)		Low ADI (*N* = 2587)	
Measurement	β (95% CI)*	FDR adjusted *P* value**	β (95% CI)	FDR adjusted *P*-value	β (95% CI)	FDR adjusted *P* value	β (95% CI)	FDR adjusted *P* value
Global Brain Measurements								
Total Cortical Surface Area (mm^2^)	0.053 (0.033, 0.074)	**<0.001**	0.054 (0.019, 0.089)	**0.006**	0.049 (0.012, 0.086)	**0.04**	0.050 (0.013, 0.086)	**0.03**
Mean Cortical Thickness (mm)	0.007 (–0.015, 0.028)	0.53						
Cortical Gray Matter Volume (mm^3^)	0.021 (0.010, 0.032)	**<0.001**	0.030 (0.010, 0.049)	**0.006**	0.015 (–0.005, 0.034)	0.20	0.023 (0.004, 0.043)	**0.04**
Subcortical Gray Matter Volume (mm^3^)	0.016 (0.003, 0.030)	**0.03**	0.019 (–0.005, 0.043)	0.15	0.024 (–0.0002, 0.049)	0.10	0.009 (–0.015, 0.033)	0.60
Cerebral White Matter Volume (mm^3^)	–0.022 (–0.033, –0.011)	**<0.001**	0.011 (–0.026, 0.049)	0.55	0.021 (–0.016, 0.058)	0.27	0.010 (–0.027, 0.047)	0.60
Adiposity Markers								
BMI *z*-scores	–0.040 (–0.063, –0.016)	**0.001**	–0.049 (–0.085, –0.001)	**0.046**	–0.042 (–0.083, –0.001)	**0.04**	–0.027 (–0.068, 0.014)	0.51
Waist Circumference (cm)	–0.037 (–0.060, –0.014)	**0.002**	–0.042 (–0.084, –0.001)	**0.046**	–0.050 (–0.089, –0.011)	**0.02**	–0.013 (–0.053, 0.026)	0.51
Waist-to-Height Ratio	–0.041 (–0.064, –0.017)	**0.001**	–0.043 (–0.085, –0.001)	**0.046**	–0.042 (–0.083, –0.001)	**0.04**	–0.017 (–0.058, 0.023)	0.51

^*^Standardized regression coefficient (95% CI) from linear mixed effects models. A positive β corresponds to larger measurements among participants breastfed for longer duration, whereas a negative β corresponds to the opposite. ** Multiple comparisons are conducted with Benjamini-Hochberg False Discovery Rate (FDR) correction. Boldface indicates significance at the corrected threshold of *P* < 0.05.

Similar results were observed from additional analyses where breastfeeding duration was dichotomized into>0 months breastfeeding vs. 0 months breastfeeding (Table S1).

### Breastfeeding duration and child adiposity markers

One category increase in breastfeeding duration was associated with 0.045 decrease in BMI *z*-scores (β (95% CI) = –0.040 (–0.063, –0.016), FDR corrected *P* = 0.001, Fig. 1B), 0.383 cm decrease in waist circumference (β (95% CI) = –0.037 (–0.060, –0.014), FDR corrected *P* = 0.002) and 0.003 decrease in WHtR (β (95% CI) = –0.040 (–0.064, –0.017), FDR corrected *P* = 0.001) (Table 2, Fig. S1B).

Additional analyses examined relationships of >0 months breastfeeding vs. 0 months breastfeeding with adiposity markers (Supporting Results, Table S1) and found similar results.

### Breastfeeding-associated effects on brain and adiposity markers, modulated by ADI

**Fig. 2:**
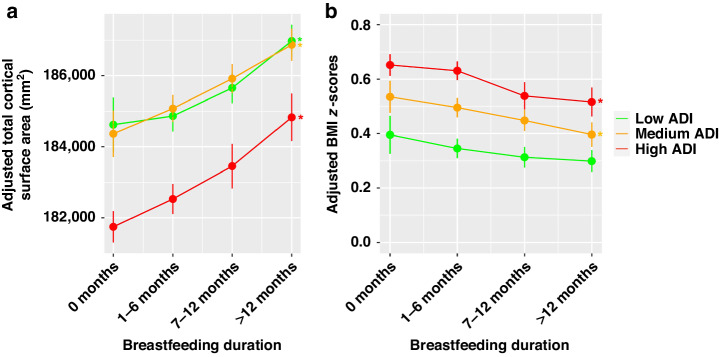
Relationships between breastfeeding duration and brain and adiposity markers in youth from neighborhoods with low, medium, and high area deprivation index (ADI). **A** Plots display association of total cortical surface area with breastfeeding duration category, in ADI tertiles, with adjustment for family ID nested within site, scanner ID, age, sex, pubertal status, race/ethnicity, family income, parental education, handedness, intracranial volume, gestational age, maternal health problems during pregnancy, child health problems at birth, maternal age at birth, and maternal alcohol or tobacco use during pregnancy. All adjustments are made within individual ADI tertiles. Points represent adjusted mean of total cortical surface area and vertical lines represent 95% CI. **B**) Plots display relationship between BMI z-scores and breastfeeding duration category, in ADI tertiles, with adjustment for family ID nested within site, pubertal status, race/ethnicity, family income, parental education, gestational age, maternal health problems during pregnancy, child health problems at birth, maternal age at birth, and maternal alcohol or tobacco use during pregnancy. All adjustments are made within individual ADI tertiles. Points represent adjusted mean of BMI *z*-scores, and vertical lines represent 95% CI. * *P* < 0.05

ADI by breastfeeding interactions were not significant for global brain measurements. Exploratory analysis was performed to investigate relationships between breastfeeding duration and global brain measures within each ADI tertile. Longer breastfeeding duration was significantly correlated with greater total cortical SA in each ADI tertile (low: (β (95% CI) = 0.050 (0.013, 0.086), FDR corrected *P* = 0.03), medium: (β (95% CI) = 0.049 (0.012, 0.086), FDR corrected *P* = 0.04), high: (β (95% CI) = 0.054 (0.019, 0.089), FDR corrected *P* = 0.006) (Table 2, Fig. 2A). Longer breastfeeding duration was significantly correlated with greater total cortical GM volume in the low-ADI group (β (95% CI) = 0.023 (0.004, 0.043), FDR corrected *P* = 0.04) and high-ADI group (β (95% CI) = 0.030 (0.010, 0.049), FDR corrected *P* = 0.006) (Table 2, Fig. S2A).

ADI by breastfeeding interactions were not observed on adiposity markers. Exploratory stratified analysis showed significant relationships between longer breastfeeding duration and smaller BMI *z*-scores in the high-ADI group (β (95% CI) = –0.049 (–0.085, –0.001), FDR corrected *P* = 0.046), and medium-ADI group (β (95% CI) = –0.042 (–0.083, –0.001), FDR corrected *P* = 0.04), but not low-ADI group (β (95% CI) = –0.027 (–0.068, 0.014), FDR corrected *P* = 0.51) (Table 2, Fig. 2B). Similar results were observed for waist circumference and WHtR (Table 2, Fig. S2B).

## Discussion

Our findings demonstrated long-term relationships between breastfeeding duration in infancy and global brain measures and adiposity markers even at 9–10 years for the entire study population, with longer breastfeeding duration being associated with greater global brain measures (i.e., cortical, and subcortical GM volumes, cortical SA) and lower adiposity markers. Furthermore, these relationships varied by SEE levels: longer breastfeeding duration was associated with lower adiposity indices in lower SEE youth (i.e., high- and medium- ADI). Longer breastfeeding duration was associated with greater cortical SA across 3 ADI levels. Our results indicate that increases in breastfeeding duration may be associated with increases in long-term associations with global cortical and subcortical GM volumes and cortical SA and decreases in long-term associations with adiposity markers. Our findings also provide preliminary support for breastfeeding associated benefits in reducing adiposity in lower SEE youth.

Longer breastfeeding duration was related to higher global brain measures in youth, most robustly in cortical SA, cortical and subcortical GM volume. These results are in line with prior studies showing positive relationship between breastfeeding duration in infancy and global brain measures years later.^2,3,8^ A binary categorization for breastfeeding (>0 months breastfeeding vs. 0 months breastfeeding) yielded similar but less significant effects because data distribution within the breastfeeding categories was skewed towards the 1–6 months duration. Our results indicate that increases in the breastfeeding duration are associated with increases in cortical SA, cortical and subcortical GM volume at 9–10 years. Studies on the ABCD dataset, have shown associations between breastfeeding duration and specific domains of cognitive performance (general ability but not executive function or memory).^26^ Consistent with our findings, this study also reported the largest effect of the association on those breastfed >12 months. Although the connection between the brain and behavior is intricate and multifaceted, a clearer picture is emerging: better cognitive performance is observed alongside larger global brain metrics linked to longer breastfeeding duration. Mothers’ breastfeeding perceptions are critical to breastfeeding duration and reports have shown many mothers find the standard medical recommendations (e.g., exclusive breastfeeding for 6 months followed by continued breastfeeding together with age-appropriate nutritious complementary foods up to 2 years) overwhelming.^60,61^ Advocacy and education aligned with lengthening breastfeeding duration, as opposed to specific limits, could improve breastfeeding rates in vulnerable women. With respect to brain results, our findings agree with other previous observations where longer breastfeeding duration was associated with better neurological and somatic outcomes with durations longer than 6 months having larger effects.^60–62^ In line with our findings, positive association of brain GM measures with duration of breastfeeding has been reported across childhood.^7^ Notably, a recent study using the ABCD data showed that longer breastfeeding duration is associated with larger global and regional cortical GM volumes, which in turn impact impulsivity personality and behavioral problems.^3^ Distinct from prior studies, we saw most robust findings in the cortical SA associated with breastfeeding duration. Taking this together with known relationships between cortical SA and adiposity markers, it further corroborates the multifaceted impact of breastfeeding on development.^63^ Our findings showed a negative correlation between breastfeeding duration and WM volume, opposite to what was reported in a prior study.^8^ It is likely that small sample size (*N* = 50) and study-specific targeted sample in pre-term infants in this prior study contributed to discrepant results. Studies of WM development in adolescents have noted a decrease in WM volume with age attributed to denser, more organized WM fibers measured as smaller volumes.^63,64^ Together with reported relationships between WM development and breastfeeding duration during early life (0–4 years),^65^ we postulate that breastfeeding is associated with healthy brain development from infancy to peri-adolescence (even after accounting for pubertal development). It has been hypothesized that breastfeeding impacts early brain development possibly by increased physical and social interactions for the infant, and improved nutrition and gut health.^64–66^ Findings from our study and others indicate that the impact of breastfeeding on brain development goes beyond infancy. The protective effects of breastfeeding on childhood obesity have been established.^67,68^ In conjunction with other published studies across childhood, our findings show that longer breastfeeding duration is associated with healthier body growth well into peri-adolescence. Although specific breastfeeding durations were recommended by different organizations, our results indicate that increases in breastfeeding duration is associated with increases in long-term associations with brain and body growth in youth.

Our most prominent finding showed that the associations of breastfeeding with brain and adiposity markers among peri-adolescents, were not uniform across SEE levels. Contrary to prior studies,^12,20,69^ we observed that youth from lower SEEs (i.e., high- and medium- ADI neighborhoods) showed significantly lower BMI *z*-scores, waist circumference and WHtR (markers of adiposity) with longer breastfeeding duration. This relationship was not significant in youth from low-ADI neighborhoods. In parallel, youth across all SEE levels demonstrated positive associations between breastfeeding duration and total cortical SA. It is worth noting that interactions of ADI by breastfeeding duration were not significant in brain and adiposity markers, thus these results need to be interpreted with caution. Mixed findings could be attributed to how various studies accounted for confounders.^70–73^ Meta-analyses have shown that the direction and strength of association between breastfeeding duration and its long-term consequences changes based on how SEE is measured or modeled in the analysis.^74,75^ For example, studies using absolute family income to measure SEE do not account for regional cost-of-living factors. The use of the nationally normed ADI measure allowed for accurate assessment of SEE in a large and diverse population. Additionally, breastfeeding duration is strongly correlated with SEE^76^ and therefore many prior studies were underpowered to disentangle the effects of SEE and breastfeeding duration on the child’s brain and body development. Leveraging a large and diverse cohort of youth in the ABCD study, our study provides critical insight into how associations between breastfeeding and brain and body development in youth vary by SEEs. Some studies have linked short breastfeeding duration among low SEE populations to the poor health outcomes to suggest that supporting longer breastfeeding duration in low SEE populations could improve child health outcomes.^77–79^ Here, we provided *direct* evidence showing stronger negative relationships between breastfeeding duration and adiposity markers in youth from lower SEE levels. Education and support aimed at increasing breastfeeding duration for lower SEE families, may partly offset their increased obesity risk.^80^

### Limitations

Our study has some limitations. This is an observational and cross-sectional study and therefore cannot establish causal mechanisms underlying these associations. Additionally, while significant, many of the effect sizes measured were small. But this was expected as development across childhood is influenced by multiple factors. Together with findings from breastfeeding intervention trials and small reduction in adiposity markers associated with breastfeeding duration observed here potentially indicate that breastfeeding alone may not curb childhood obesity.^81^ We acknowledge that the reported breastfeeding durations may be subject to maternal recall bias 9–10 years after its occurrence. Additionally, breastfeeding length did not account for exclusive breastfeeding or combination feeding in the first 6 months. Confounders included in this analysis were limited to those available within the ABCD database.

## Conclusion

This is the largest multi-site study investigating the relationships between breastfeeding duration and brain structure and adiposity markers in 9–10-year-old youth. There were positive long-term associations of breastfeeding duration with global brain measures (i.e., cortical SA, cortical and subcortical GM volumes), and negative associations with adiposity markers. Youth from lower SEEs had stronger negative associations between breastfeeding duration and adiposity markers. These results suggest that long-term associations of breastfeeding with brain and body development, persist into peri-adolescence for the entire study population, and breastfeeding associated reductions in adiposity markers are larger for youth from lower SEEs. Interventions and social policies designed to increase overall breastfeeding rates and duration has the potential to reduce child health disparities based on SEEs.

## Supplementary information

Supplementary information

## Data Availability

Data used in the preparation of this article were obtained from the Adolescent Brain Cognitive Development (ABCD) ® Study (https://abcdstudy.org), held in the NIMH Data Archive (NDA). This is a multisite, longitudinal study designed to recruit more than 10,000 children age 9–10 and follow them over 10 years into early adulthood. The ABCD data repository grows and changes over time. The ABCD data used in this report came from NIMH Data Archive Digital Object Identifier (DOI). DOI can be found at 10.15154/1528510. The ABCD Study® is supported by the National Institutes of Health and additional federal partners under award numbers U01DA041048, U01DA050989, U01DA051016, U01DA041022, U01DA051018, U01DA051037, U01DA050987, U01DA041174, U01DA041106, U01DA041117, U01DA041028, U01DA041134, U01DA050988, U01DA051039, U01DA041156, U01DA041025, U01DA041120, U01DA051038, U01DA041148, U01DA041093, U01DA041089, U24DA041123, U24DA041147. A full list of supporters is available at https://abcdstudy.org/federal-partners.html. A listing of participating sites and a complete listing of the study investigators can be found at https://abcdstudy.org/consortium_members/. ABCD consortium investigators designed and implemented the study and/or provided data but did not necessarily participate in the analysis or writing of this report. This manuscript reflects the views of the authors and may not reflect the opinions or views of the NIH or ABCD consortium investigators.
