# Supplementary material for: Breastfeeding duration and brain-body development in 9–10-year-olds: modulating effect of socioeconomic levels
Source: Pediatr Res. 2024 Jun 15;97(1):378–86. doi: 10.1038/s41390-024-03330-0 (PMC11798855; doi:10.1038/s41390-024-03330-0)
Supplement: Supplementary file 1 — Supplementary information [file 41390_2024_3330_MOESM1_ESM.pdf]

Supporting Information Text

Supporting Methods

Our final sample included 6501 families, which are distributed between sites as follows:

Site #	# of families
Site 1	210
Site 2	255
Site 3	403
Site 4	394
Site 5	222
Site 6	384
Site 7	203
Site 8	206
Site 9	272
Site 10	379
Site 11	271
Site 12	324
Site 13	382
Site 14	264
Site 15	224
Site 16	655
Site 17	372
Site 18	249
Site 19	179
Site 20	347
Site 21	306

Covariates

Race and/or ethnicity were determined based on caregiver-reported questionnaire responses (e.g., American Indian/ Native American, Asian Indian, Black or African American, Chinese, Filipino, Guamanian, Hispanic or Latino, Japanese, Korean, Native Hawaiian, other Asian, other Pacific Islander, Samoan, Vietnamese, White, and other race) and grouped into five categories: Asian, Black non-Hispanic, Hispanic or Latino, White non-Hispanic, and Other, where "Other" included youth identified as Native American or American Indian, Native Hawaiian, Pacific Islander (Guamanian, Samoan, or other Pacific Islander), and multiple races (Asian, non-Hispanic Black, non-Hispanic White). Pubertal stage was self-reported by parents using the pubertal development scale, is then reduced to three levels (pre-pubertal, early pubertal, mid-pubertal and above), by combining for all levels mid-puberty and above as one level, in order to pool small samples in late- and post-puberty. Gestational age was derived as 40 weeks for children not born premature, and otherwise by subtracting the number of weeks premature from 40. Maternal health problem(s) during pregnancy was modeled as a binary variable (1 for any problem such as severe nausea or vomiting, heavy bleeding, pre-eclampsia, eclampsia or toxemia, gall bladder attack, proteinurea, rubella, severe anemia, maternal diabetes, urinary tract infections, pregnancy-related high blood pressure, placenta problems, an accident or injury requiring medical care, otherwise 0). Infant's health problem(s) at birth was modeled as a binary variable (1 for any problem including following, blue at birth, slow heartbeat, did not breath at first, convulsions, jaundice needing treatment, required oxygen, required blood transfusion, Rh incompatibility, otherwise 0). Maternal age at birth was self-reported by the caregiver. Maternal alcohol/tobacco use during pregnancy was

coded as a binary variable indicating self-reported use of either substance, either prior to or after knowing of pregnancy.

Analysis
Linear mixed effects models were conducted for analysis of correlations of breastfeeding with brain and adiposity markers, with breastfeeding coded as a binomial variable (0 months breastfeeding vs. >0 months breastfeeding). Family ID, nested within site, were modeled as random intercepts in models of adiposity markers; and Family ID, nested within scanner ID, were modeled as random intercepts for models of brain measurements. Covariates included age, sex assigned at birth, pubertal status, race/ethnicity, family income, and highest parental education, maternal age, health problems during pregnancy, health problems at birth, gestational age, and maternal alcohol/tobacco use. Age and sex were not included as covariates for models with BMI *z*-score as a dependent variable. Handedness was included in models of brain measurements and intracranial volume was in added in volumetric models.

Supplementary analyses were conducted in R. Linear mixed effects models were fit using the *lme4* package, using Satterthwaite's method to calculate *P*-values in the *lmerTest* package. Standardized betas were reported, with 95% Wald confidence intervals were calculated based on the local curvature of the likelihood surface. Tests of significance (2-tailed) were corrected for multiple comparisons using the Benjamini-Hochberg false discovery rate (FDR) correction, with $P<0.05$ as the corrected threshold for significance. Cohen's *d* effect sizes were calculated from least-squares means, model residual standard deviation (SD), and residual degrees-of-freedom using the *emmeans* package.

Supporting Results

Breastfeeding and Youth Global Brain Measurements
Results for monotonic relationships between breastfeeding duration and *other* global brain measures were displayed in Figure S1A. Breastfeeding of >0 months (vs. 0 months) was associated with smaller cerebral white matter volume (β (95% CI) = -0.055 (-0.084, -0.026), FDR corrected $P<0.001$). Although results were not significant, breastfeeding >0 months vs. 0 months demonstrated a similar data pattern as breastfeeding duration in relation to total cortical gray matter volume (β (95% CI) =0.026 (-0.002, 0.054), FDR corrected $P=0.12$) and surface area (β (95% CI) =0.052 (-0.001, 0.104), FDR corrected $P=0.12$). For details see Table S1.

Breastfeeding and Youth Adiposity Markers
Results for monotonic relationships between breastfeeding duration and other adiposity markers were displayed in Figure S1B. Breastfeeding of >0 months (vs. 0 months) was associated with smaller waist circumference (β (95% CI) = -0.069 (-0.128, -0.011), FDR corrected $P=0.03$) and smaller waist-to-height ratio (β (95% CI) = -0.073 (-0.132, -0.013), FDR corrected $P=0.03$).

Breastfeeding and Youth Global Brain Measures and Adiposity Markers in Area Deprivation Index (ADI) Tertiles
Figure S2A and S2B displayed relationships between breastfeeding duration and other global brain measures and adiposity markers in youth from neighborhoods with low, medium, and high ADI.

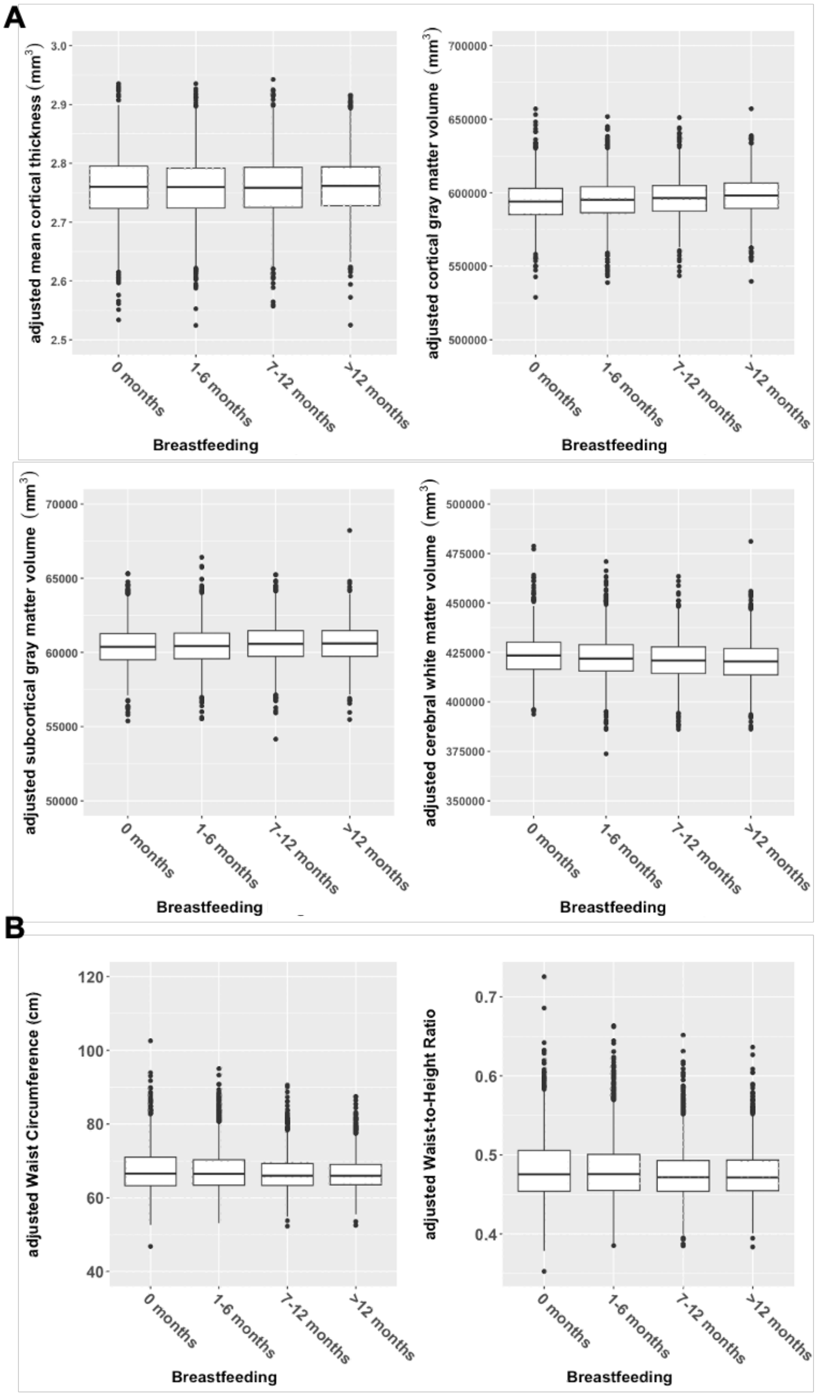

Figure S1. Relationships between breastfeeding duration and brain and adiposity markers. A). Boxplots display distributions of mean cortical thickness, total cortical gray matter volume, subcortical gray matter volume, and cerebral white matter volume (adjusting for family ID nested within scanner ID, handedness, intracranial volume, age, sex, pubertal status, race/ethnicity, family income, parental education, gestational age, maternal health problems during pregnancy, child health problems at birth, maternal age at birth, and maternal alcohol or tobacco use during pregnancy) separated by breastfeeding duration category. Analysis of breastfeeding and cortical thickness does not adjust for intracranial volume. B). Boxplots display distributions of waist circumference and waist-to-height ratio (adjusting for family ID nested within site, pubertal status, race/ethnicity, family income, parental education, gestational age, maternal health problems during pregnancy, child health problems at birth, maternal age at birth, and maternal alcohol or tobacco use during pregnancy) separated by breastfeeding duration category.

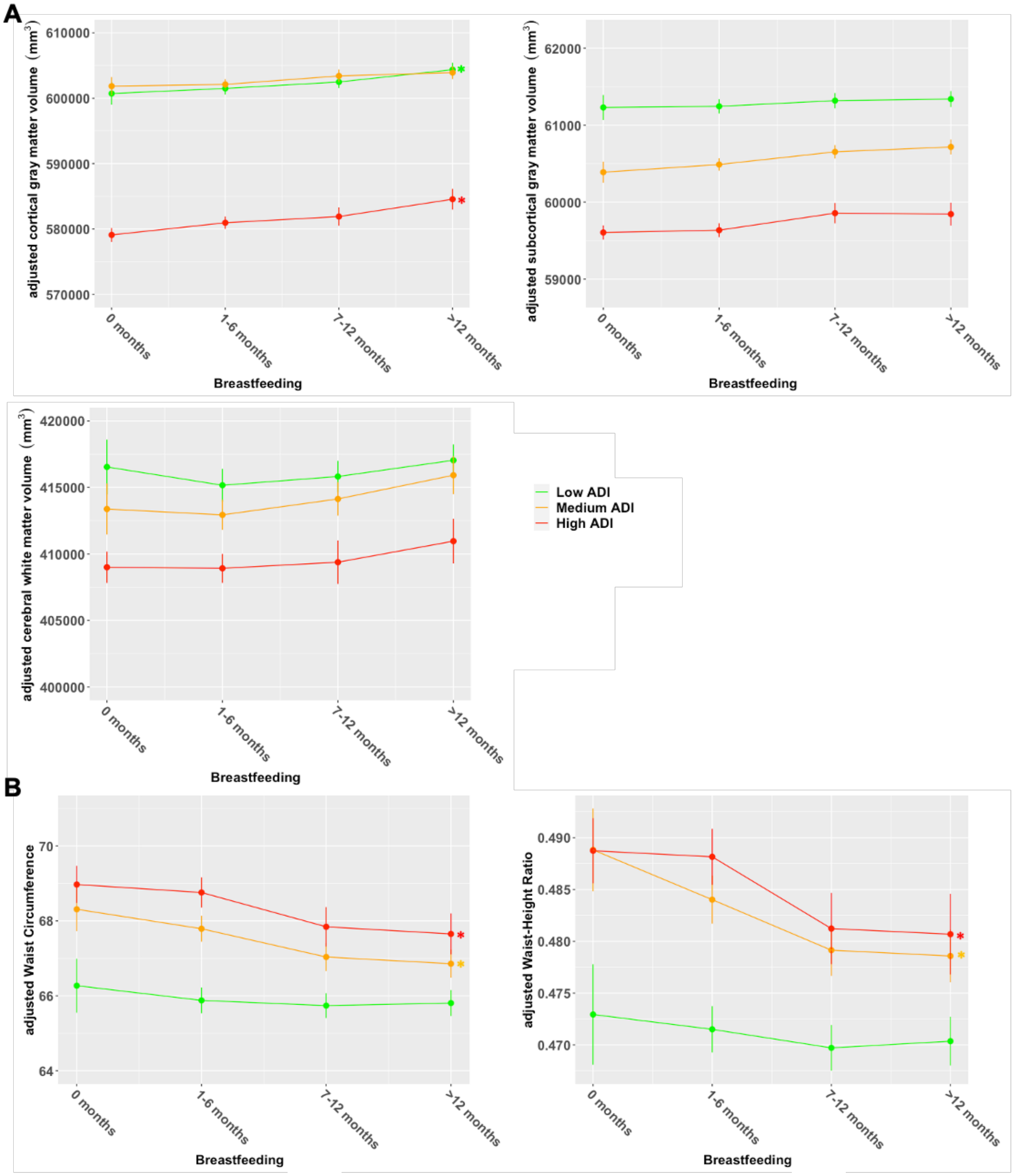

Figure S2. Relationships between breastfeeding duration and global brain measures and adiposity markers in youth from neighborhoods with low, medium, and high area deprivation index (ADI). A) Plots display relationship of total cortical gray matter volume, subcortical gray matter volume, and cerebral white matter volume with breastfeeding duration category and ADI (in tertiles), adjusting for family ID nested within site, scanner ID, age, sex, pubertal status, race/ethnicity, family income, parental education, handedness, intracranial volume, gestational age, maternal health problems during pregnancy, child health problems at birth, maternal age at birth, and maternal alcohol or tobacco use during pregnancy. B) Plots display relationship of waist circumference and waist-to-height ratio with breastfeeding duration category and ADI (in tertiles), with adjustment for family ID nested within site, age, sex, pubertal status, race/ethnicity, family income, parental education, gestational age, maternal health problems during pregnancy, child health problems at birth, maternal age at birth, and maternal alcohol or tobacco use during pregnancy.

* FDR Adjusted P<0.05

Table S1. Associations between Breastfeeding >0 months (vs. 0 months Breastfeeding) and Global Brain Measurements and Adiposity Markers

**Measurement**	**Std β**	**95% CI**	**Cohen's *d***	***P*-value**	**FDR adjusted *P*-value**
**Global Brain Measurements**					
Total Cortical Surface Area	0.052	(-0.001, 0.104)	0.101	0.05	0.12
Mean Cortical Thickness	-0.008	(-0.063, 0.047)	-0.012	0.77	0.77
Cortical Gray Matter Volume	0.026	(-0.002, 0.054)	0.082	0.07	0.12
Subcortical Gray Matter Volume	0.019	(-0.015, 0.054)	0.052	0.27	0.34
Cerebral White Matter Volume	-0.055	(-0.084, -0.026)	-0.222	<0.001	**<0.001**
**Adiposity Markers**					
BMI *z*-scores	-0.057	(-0.117, 0.003)	-0.087	0.06	0.06
Waist Circumference	-0.069	(-0.128, -0.011)	-0.101	0.02	**0.03**
Waist-to-Height Ratio	-0.073	(-0.132, -0.013)	-0.104	0.02	**0.03**
